# Serum cytokines as a biomarker for immune checkpoint inhibitor toxicity in patients with pleural mesothelioma

**DOI:** 10.3389/fimmu.2024.1480183

**Published:** 2024-12-02

**Authors:** Saima Jamil Farooqi, Zhi Zhao, Åsa Kristina Öjlert, Solfrid Thunold, Hedvig Vidarsdotter Juul, Maria Moksnes Bjaanæs, Henrik Horndalsveen, Hanne Marte Gjertsen Nymoen, Åslaug Helland, Vilde Drageset Haakensen

**Affiliations:** ^1^ Department of Oncology, Oslo University Hospital, Oslo, Norway; ^2^ Department of Cancer Genetics, Oslo University Hospital, Oslo, Norway; ^3^ Oslo Centre for Biostatistics and Epidemiology (OCBE), Institute of Basic Medical Sciences, Faculty of Medicine, University of Oslo, Oslo, Norway; ^4^ Cellular Therapy, Division of Cancer Medicine, Oslo University Hospital, Oslo, Norway; ^5^ University of Oslo, Institute of Clinical Medicine, Oslo, Norway

**Keywords:** pleural mesothelioma, immunotherapy, biomarkers, cytokines, checkpoint inhibition blockade

## Abstract

**Background:**

Pleural mesothelioma (PM) is a rare cancer with a dismal prognosis. Dual immune checkpoint inhibitors have improved overall survival, but the rate of immune-related adverse events (irAEs) is high. Serum cytokines reflect systemic immune reactions and may serve as biomarkers for irAEs.

**Patients and methods:**

Patients with pleural mesothelioma treated with nivolumab and ipilimumab with or without UV1 vaccine in the NIPU study were included. Serum cytokine levels were measured by Bio-Plex Pro Human Cytokine Screening 48-Plex Panel Assay. Correlations between cytokine levels and irAEs were analyzed by generalized linear mixed models to identify potential diagnostic and predictive biomarkers.

**Results:**

Higher levels of MIG, eotaxin, MIP-1α, IP-10, TNF-α, MIP-1β, IL-4, MIF, IL-16, IL-2RA, SCGF.β and PDFG-BB at baseline are associated with increased risk of developing one or more irAEs. In particular, higher baseline levels of MIG are positively associated with thyroiditis and hypophysitis, and elevated levels of IP-10 and MIG to dermatitis. During the course of treatment, higher levels of MIG, eotaxin, MIF, TNF-α, MIP-1β, IL-4 and IL-16 are associated with an ongoing irAE. We found both predictive and diagnostic value of MIF with fatigue and of eotaxin with both colitis and pneumonitis. Higher levels of CTACK is associated with a lower risk of developing hepatitis, both before and after treatment.

**Conclusions:**

Elevated levels of certain cytokines, both before and after onset of treatment, correlate with specific irAEs in PM patients receiving ICIs. These cytokines may be used as biomarkers to predict and detect irAES.

## Introduction

Pleural mesothelioma (PM) is a rare and aggressive malignancy, originating from the pleural lining. Until recently, the only systemic treatment available was platinum-based chemotherapy combined with pemetrexed, with or without bevacizumab. The combination of dual immune checkpoint inhibitors (ICIs), nivolumab (anti PDL-1) and ipilimumab (anti CTLA-4), has demonstrated significantly improved overall survival compared to standard chemotherapy (1) and has been approved by the FDA and EMA as first line treatment for patients with PM (2). Dual ICIs has prolonged overall survival but is associated with a high rate of immune-related adverse events (irAEs) with 49% of patients experiencing grade 1-2 and 30% of patients experiencing grade 3-4 events (1). The combined toxicity of ipilimumab and nivolumab is well known. The safety and immunological response of the UV1 vaccine, when given both as monotherapy and in combination with ICI, have previously been investigated (3, 4). Together, these studies have shown that UV1 is generally safe and well tolerated. Common adverse events related to UV1 include pruritus, erythema, fatigue, diarrhea, pain, and rash. Early diagnosis of irAEs and onset of corticosteroids is essential to prevent progression to higher grade leading to permanent discontinuation of treatment, morbidity and possibly mortality.

Predictive biomarkers can help identify patients at higher risk of developing irAEs, allowing for closer monitoring, early intervention, and identification of mechanisms underlying irAEs. Biomarkers represent a critical component of the ongoing efforts to make immunotherapy more effective and safer for patients with cancer.

Cytokines are small proteins or signaling molecules involved in intercellular interaction and communication. They are released by activated cells, including immune cells, upon stimuli and are engaged in innate as well as adaptive inflammatory host defenses, cell growth, differentiation, cell death, angiogenesis, and overall homeostasis (5). Changes in cytokine levels can provide insights into the immune response. Cytokines are detectable in peripheral blood, suitable for dynamic assessment, and therefore they can serve as potential biomarkers for prediction and early detection of irAEs.

In this report, we have studied serum cytokine levels in patients with PM treated with double ICIs, with or without the UVI vaccine in order to investigate 1) whether baseline levels of certain cytokines may be associated with a patient’s risk of developing a specific irAE 2) whether changes in cytokine levels are associated with the detection of irAEs, potentially serving as a diagnostic biomarker.

## Patients and methods

### Patient population

Patients analyzed in this study were included in the NIPU study, a randomized, multi-center, open-label, proof of concept study comparing the efficacy and safety of nivolumab and ipilimumab with or without the UV1 telomerase vaccine, in patients with inoperable PM after first-line platinum-based chemotherapy (6).

In total 118 patients were included and randomized to ipilimumab and nivolumab alone (ARM B) or in combination with UV1 vaccine (ARM A). The patients were treated with nivolumab 240 mg q2w + ipilimumab 1 mg/kg q6w until progression or unacceptable toxicity, for a maximum of 2 years. Patients randomized to the intervention arm (arm A) received 8 injections (13 weeks) of 300 μg UV1. UV1 is administered intradermally with 75 μg of granulocyte-macrophage colony-stimulating factor (GM-CSF) as an adjuvant.

A total of 74 patients were selected for cytokine analysis, with 34 in Arm A and 40 in Arm B (**Table 1A**). Due to practical constraints, sample processing was conducted in two rounds. In the first round, 27 patients were randomly selected from the trial participants who had at least two time points available, providing a representative baseline for cytokine levels across the cohort. In the second round, as more patients enrolled and additional assay kits became available, we focused on patients who had experienced irAEs, adding 47 samples to increase representation and enhance the study’s power to detect associations between cytokine levels and specific irAEs. Consequently, the proportion of patients with irAEs in the cytokine analysis cohort is higher than in the overall study population (7).

**Table 1A:** Number of patients analyzed and number of irAEs experienced, by arm and grade.

	Total	ARM A (with UV1)	ARM B (without UV1)
No of patients	74	34 (46%)	40 (54%)
Patients with irAEs	65	31 (48%)	34 (52%)
irAEs	121	58 (48%)	63 (52%)
Grade 1-2 irAEs	107	53 (50%)	54 (50%)
Grade 3 irAEs	14	6 (43%)	8 (57%)
Patients with a single irAE	21	6 (29%)	15 (71%)
Patients with multiple irAEs	44	25 (57%)	19 (43%)
Patients with grade 1/2 irAEs	51	25 (49%)	26 (51%)
Patients with grade 3 irAEs	14	6 (43%)	8 (57%)

During statistical analyzes five patients were excluded due to lack of screening samples. Information about side effects and the use of glucocorticoids (GCs) was collected from the electronic case report forms and the patient records. None of the selected patients were taking GCs at the time of screening; however, some patients had already initiated GC therapy by the time serum samples were collected, subsequent to reporting side effects.

### Serum samples

Serum samples were collected at baseline, at the first three subsequent evaluations (week 6/7, 12/13, 18/19) and at end of treatment. We collected baseline and week 6/7 serum samples from all selected patients. However, not all patients had undergone evaluation beyond week 6/7.

Serum was collected in a serum gel tube and left to clot for 30-120 minutes, prior to centrifugation at 2500 g for 15 min at room temperature. They were then aliquoted and frozen at −80°C until thawn for biomarker analyses.

### Multiplex cytokine/chemokine assay

The commercially available Bio-Plex Pro Human Cytokine Screening 48-Plex Panel Assay (Catalog Number 12007283, Bio-Rad Laboratories) allowed the simultaneous determination of 48 cytokines, in each well of a 96-well plate. The assay was chosen due to its comprehensive coverage of cytokines, which we believed would provide insights into immune-related adverse event. The Bio-Plex assays were performed on serum specimens according to the manufacturers’ instructions. Briefly, 50 μl of diluted magnetic beads were placed in the wells and washed two times with Bio-Plex Wash Buffer. Then, the standard samples and controls were pipetted into the respective wells and incubated for 30 min on the shaker at 850 rpm at room temperature. This incubation was followed by a three-fold wash step, the addition of the diluted detection antibody, and an incubation of 30 min. After another wash step, streptavadin-phycoerythrin was added and incubated for 10 min. Finally, the plate was washed three times and resuspended in 125 μl assay buffer and incubated for 30 seconds. All incubation steps were performed at room temperature. Measurements were carried out on a Bio-Plex 200.

### Adverse events

Adverse events were reported according to the Common Terminology Criteria for Adverse Events (CTCAE 5.0). Data cut-off for the first 2 batches was February 2022 and for the last 4 batches December 2022.

The most common irAEs related to dual ICI were selected for further analysis: Fatigue, dermatitis, colitis, pneumonitis, hypophysitis, thyroiditis and hepatitis (8). The irAEs allergic reaction, arthralgia, pancreatitis and nephritis are less common but observed in our patients and were also included in the analyses (**Table 1B**). For the 11 irAEs of interest, we evaluated serum cytokine levels before and during treatment. For each irAE analyzed, the control group consisted of patients who did not develop that specific irAE. Patients who experienced other irAEs were still included in the control group for a given irAE, as long as they did not develop the irAE being analyzed.

**Table 1B:** Summary of number of irAEs experienced by Type and Severity Grade.

Type of AE	Grade 1	Grade 2	Grade 3
Pneumonitis	16	6	8
Dermatitis	29	16	12
Hepatitis	7	5	1
Thyroiditis	9	6	4
Pancreatitis	5	2	2
Fatigue	15	13	3
Arthralgia	6	6	1
Colitis	14	6	5
Hypophysitis	4	1	2
Nephritis	4	3	1
Allergic reaction	12	5	5

### Statistical analysis

#### Data preprocessing

The adverse events were analyzed as binary outcome data, i.e., with occurrence of an adverse event or not. Cytokine data that was out of range, whether too high or too low, was excluded, resulting in 26 remaining cytokines. To remove the batch effect, we standardized the cytokine data within each plate, i.e., the levels of each cytokine from all samples run in one batch were transformed into z-scores separately.

For each patient, we used the data of all available cytokines before the treatment as baseline levels. After onset of treatment, we only considered the cytokine data at the time of the irAE or at week 6/7 if there was no irAE. This allowed us to take into account the baseline heterogeneity between patients and better investigate whether any cytokines are associated with the occurrence of an adverse event of interest.

#### Mixed models for analyzing cytokines

To identify cytokines associated with the occurrence of irAEs and take into account the correlations of repeatedly measured cytokines from each patient, we applied generalized linear mixed models by L1-penalized estimation (glmmLasso) (9) for each adverse event, since classic generalized linear models do not yield stable estimates when there are many cytokines. To account for uncertainty for each adverse event outcome model, we used the Bootstrap method to run the glmmLasso 100 times and determine if an identified cytokine has nonzero coefficient in at least 10% of all the repeats. For each identified cytokine, we reported the conditional mean effect (ME) to measure the effect of this cytokine and the corresponding standard deviation (SD) to measure uncertainty. If an SD is larger than the corresponding absolute ME, we do not conclude an association of the cytokine with the irAE because of large uncertainty of the estimated effect. A cytokine with positive ME value indicates that a higher level of the cytokine at baseline is associated with a higher risk of developing the adverse event, while a negative ME value indicates the opposite.

Since we are interested in how an irAEs affects changes of cytokines levels, we applied the glmmLasso by including all interactions between the 26 cytokines and evaluation time variable (interaction term Cytokine: Time) as predictors. An interaction Cytokine: Time with positive ME value indicates that an increase in the cytokine level after treatment is associated with a higher likelihood of the irAEs.

To assess whether prednisolone would impact cytokine levels, in the glmmLasso method we added the interaction between cytokine, time and prednisolone for modeling each irAE. A positive ME indicates that prednisolone treatment would lead to an additional increase in the specific cytokine level associated with that irAE.

## Results

A total of 74 patients were analyzed, ARM A (with UV1) had 34 patients (46%), and ARM B (without UV1) had 40 patients (54%). A total of 65 patients (88%) experienced immune-related adverse events (irAEs), with 121 irAEs reported overall: 58 (48%) in ARM A and 63 (52%) in ARM B.

Most irAEs were mild (Grades 1-2), with 107 occurrences evenly split between the groups. There were 14 Grade 3 irAEs, with ARM A having 6 (43%) and ARM B having 8 (57%). Single irAEs were reported in 21 patients, predominantly in ARM B (71%), while multiple irAEs affected 44 patients, more common in ARM A (57%) (**Table 1A**) 74 patients experienced a total of 121 of the irAEs of interest during the time the data was collected. 113 adverse events were grade 1-2 and 15 were grade 3. While there were more patients in Arm A who experienced multiple irAEs, there was no major difference in the overall number of adverse events experienced by patients in Arm A (48%) versus Arm B (52%) (**Table 1A**).

**Figure 1:**
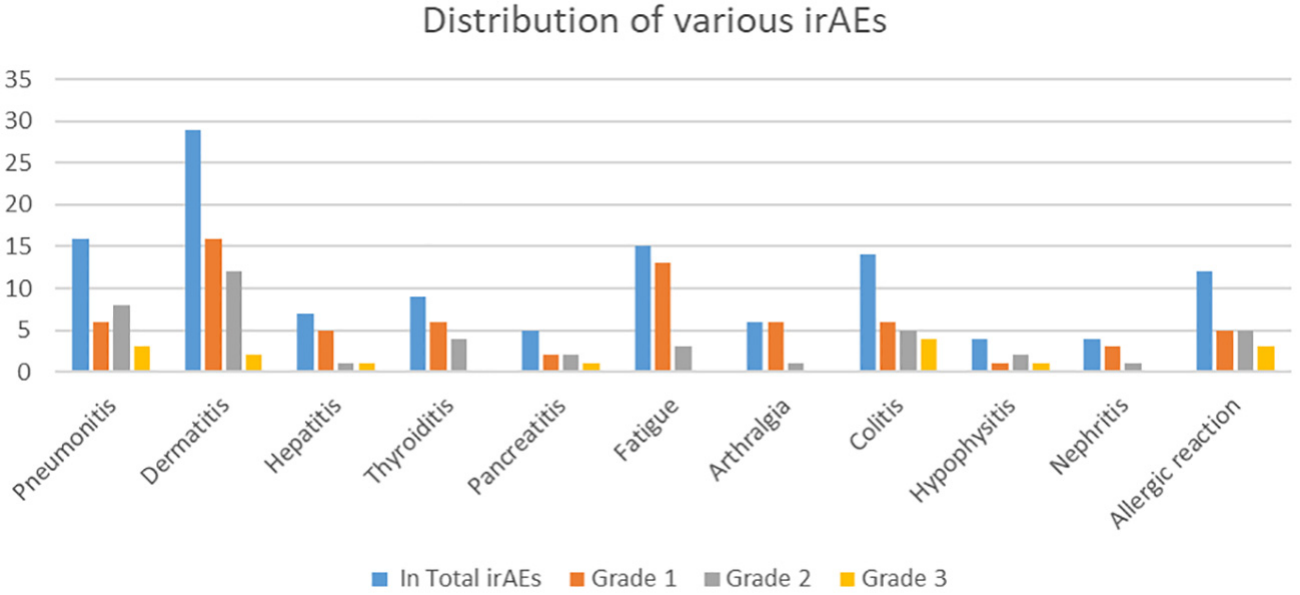
Distribution of various irAEs by grade, with the Y-axis representing the number of irAEs and the X-axis showing the irAE types.

The most common immune-related adverse events (irAEs) across all grades included dermatitis, fatigue, and pneumonitis. Dermatitis was the most frequent, with 29 AE Grade 1, 16 AE Grade 2, and 12 AE Grade 3 occurrences. Fatigue and pneumonitis followed, primarily in lower grades. Other notable irAEs included colitis, thyroiditis, and allergic reactions, each with a range of severity. Grade 3 events were less frequent, with dermatitis, colitis, and allergic reactions having the highest numbers at this severity level (**Table 1B**). There were no grade 4 or 5 irAEs in this population of patients (**Tables 1A**, **B**; **Figure 1**).

**Figure 2:**
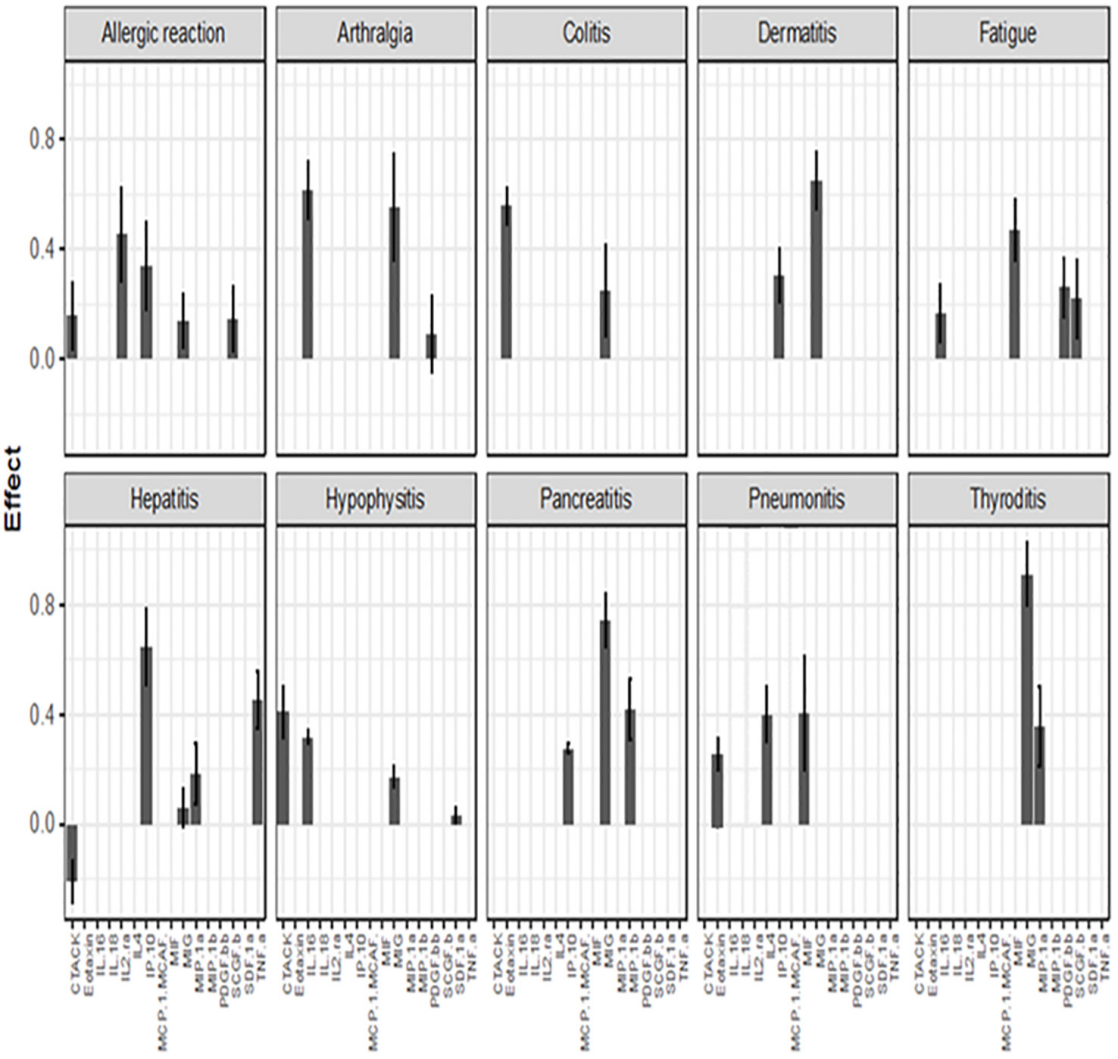
Associations between baseline cytokine levels and future development of irAEs. Each figure panel shows the effect of the identified cytokine. The height of a bar is the conditional ME of the identified cytokine and the corresponding error bar shows the SD of the cytokine’s effect. A positive ME indicates that high baseline level is associated with increased risk of developing the irAE, while a negative value suggests a protective association.

**Figure 3:**
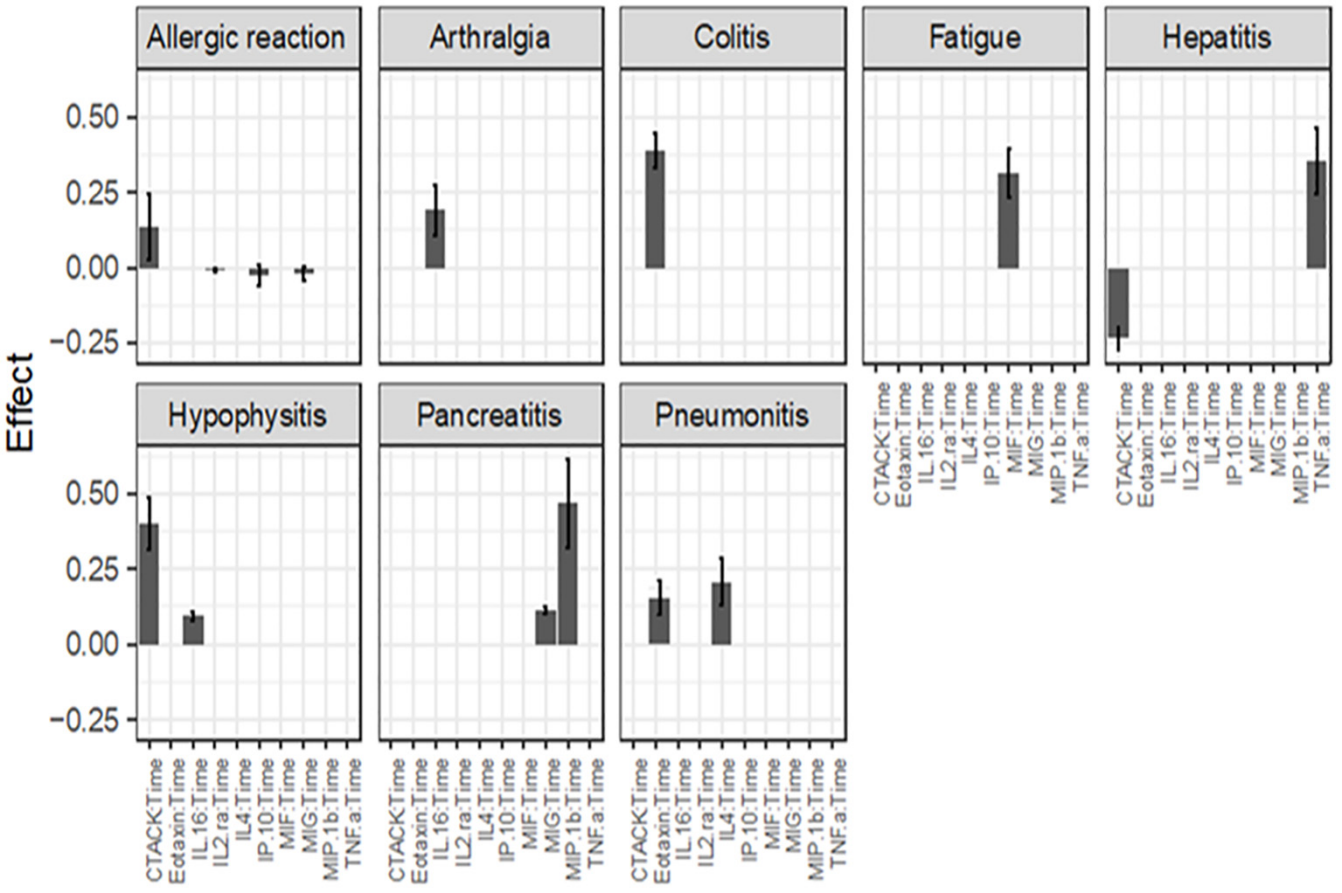
Association between cytokine level and ongoing irAEs. Each figure panel shows the conditional ME and SD of the identified Cytokine: Time interaction corresponding to an irAE. Association is drawn if the absolute ME of a Cytokine: Time interaction is larger than its corresponding SD.

After using the glmmLasso to model all irAEs separately, we found that higher baseline expression of 13 cytokines (MIG, eotaxin, MIP-1α, IP-10, TNF-α, CTACK, MIP-1β, IL-4, MIF, IL-16, IL-2RA, SCGF.β, and PDFG-BB) is associated with at least one irAEs (**Table 2**; **Figure 2**). During the course of treatment, elevated levels of MIG, eotaxin, MIF, TNF-α, MIP-1β, IL-4 and IL-16 is associated with at least one ongoing irAE (**Table 3**; **Figure 3**).

**Table 2:** Associations between baseline cykotine levels and future development of irAEs, the number is the mean effect (ME) and the number in parentheses is the standard deviation (SD). The ME represents the estimated average impact of a cykotine on the irAEs. The SD provides a measure of the uncertainty associated with the estimated ME. A positive ME for a cykotine suggests that higher level of the cykotine before treatment is associated with higher risk of developing the irAEs, negative ME suggest the opposite.

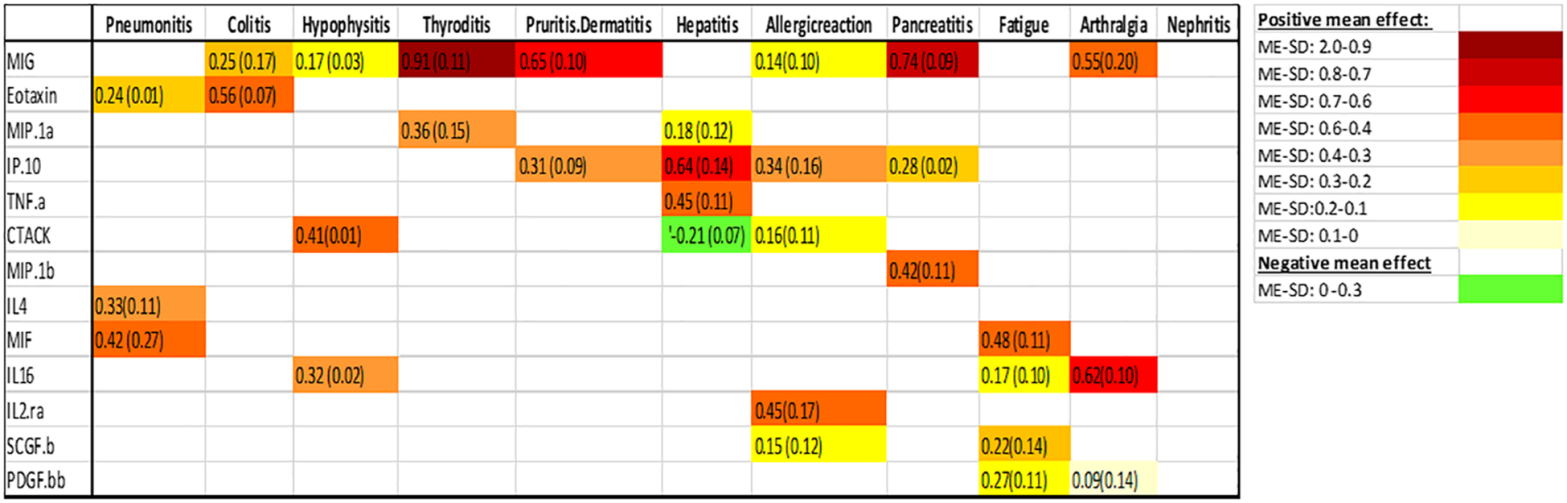

**Table 3:** Association between cytokine level and an ongoing irAE at any given point in time.

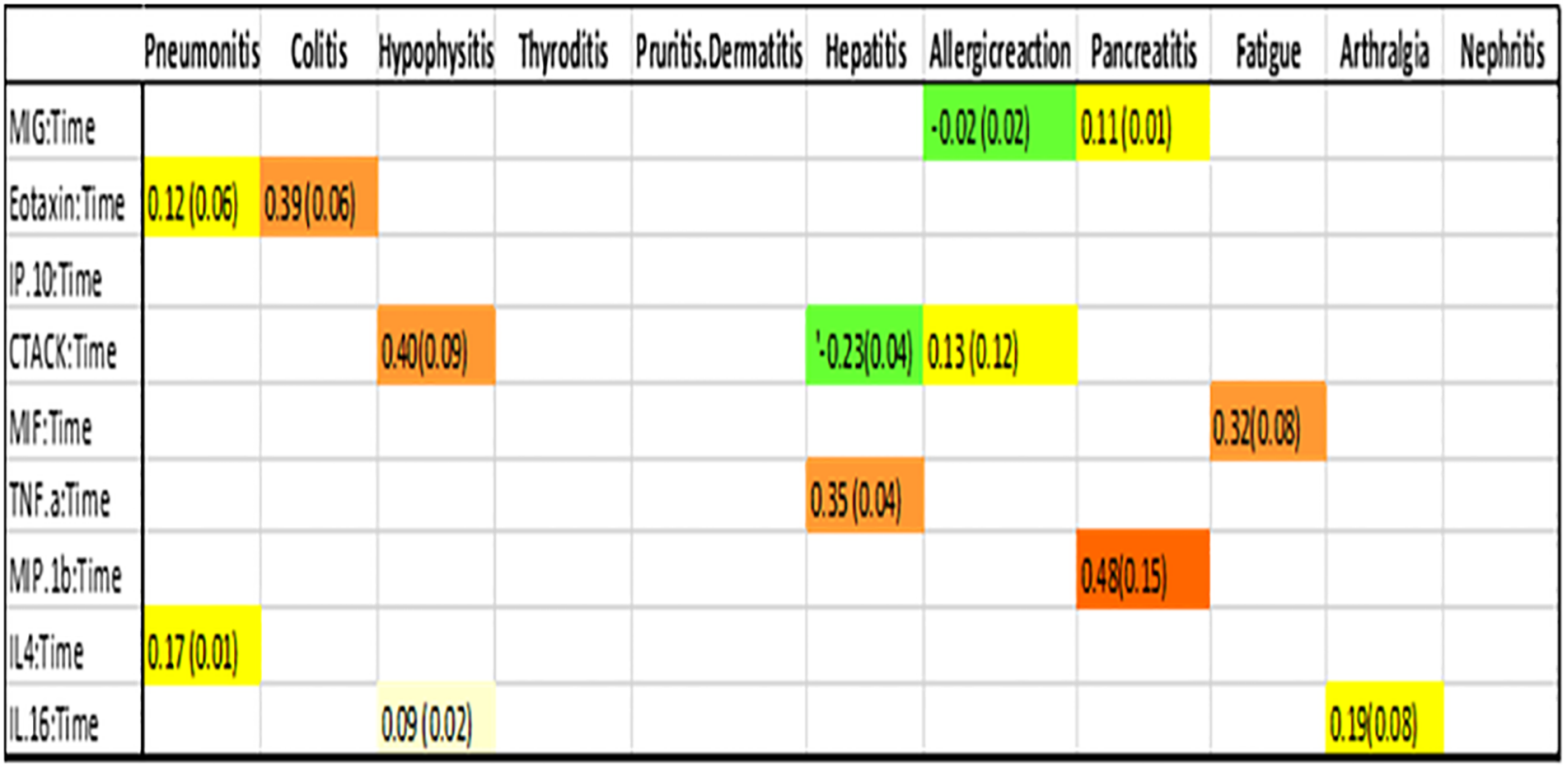

During treatment an increase in the cytokine is associated with the irAEs. If the ME is positive, it means as the cytokine increases over time, it is more likely that the patient has the irAEs. If the ME is negative, it indicates that increasing cytokine levels reduce the likelihood of the patient experiencing the irAEs.

The color intensity of the box correspond to the value of the ME and SD, with darker shades signifying higher ME and smaller SD (**Table 2**).

Associations between baseline cytokine levels and future development of irAEs, the number is the mean effect (ME) and the number in parentheses is the standard deviation (SD). The ME represents the estimated average impact of a cytokine on the irAEs. The SD provides a measure of the uncertainty associated with the estimated ME. A positive ME for a cytokine suggests that higher level of the cytokine before treatment is associated with higher risk of developing the irAEs, negative ME suggest the opposite.

**Figure 2** (panel hepatitis) shows that four cytokines have baseline levels associated with the future development of hepatitis; MIP-1α, (ME 0.18, SD 0.12), TNF-α (ME 0.45, SD 0.11), Interferon gamma-induced protein 10 (IP-10) (ME 0.64, SD 0.14) and cutaneous T cell-attracting chemokine (CTACK) (ME -0.21, SD 0.07). The low ME (0.18) and high SD (0.14) of MIP.1α makes an association between this cytokine and future hepatitis uncertain. The cytokine CTACK has a negative ME, meaning that high baseline levels of the cytokine CTACK may reduce the risk of development of hepatitis. **Figure 3** (panel hepatitis) shows that serum levels of CTACK during treatment are negatively correlated with hepatitis (ME -0.23, SD 0.04). **Figure 3** also indicates that during treatment, an increase in TNF-α is associated with concurrent hepatitis (ME 0.35, SD 0.04).

For the development of an allergic reaction, **Figure 2** shows that the baseline levels of five cytokines are associated. Monokine induced gamma interferon (MIG) (ME=0.14, SD=0.10), CTACK (ME=0.16, SD=0.11), stem cell growth factor beta (SCGF.b) (ME=0.15, SD=0.12), IP.10 (ME 0.34, SD 0.16) and IL2 (ME 0.45, SD 0.17). IP.10 and IL2RA has lower SD compared to the ME, suggesting a more reliable estimated average impact of these two cytokines on allergic reaction.

There were no cytokines identified to be associated with the irAE nephritis.

**Figure 4:**
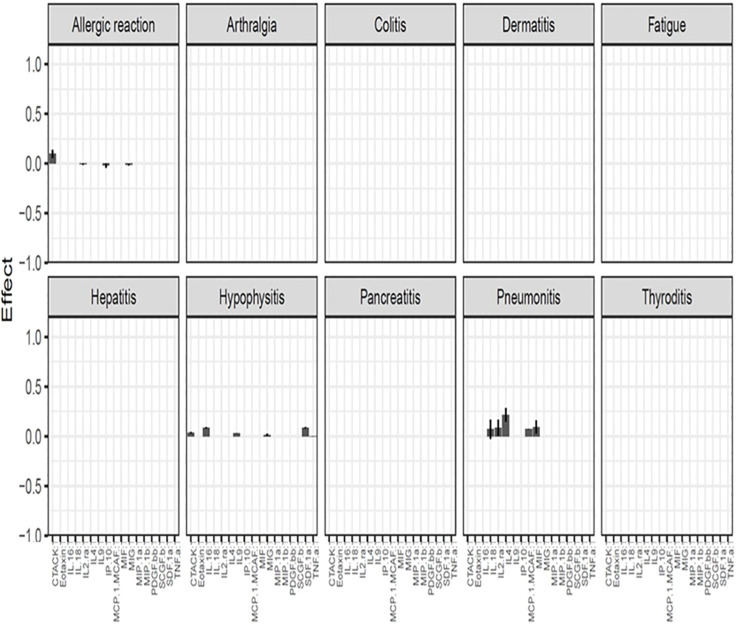
Effect of prednisolone treatment on cytokine levels in various irAEs. An association is significant if the ME of the cytokine exceeds its corresponding SD. A positive ME indicates that prednisolone results in a further increase in the specific cytokine compared to patients who are not receiving prednisolone for the particular irAE.

We conducted an analysis to assess the effect of prednisolone administration on cytokine levels in different irAEs, given that some patients had initiated prednisolone treatment before serum sample collection (**Table 4**). As shown in **Figure 4**, prednisolone influences IL-4 levels in pneumonitis, GRO-α levels in hypophysitis, and CTACK levels in allergic reactions, resulting in a slight further increase in the cytokine level for the respective irAE, compared to patients not receiving prednisolone. Beyond these observations, prednisolone did not exhibit a measurable impact on the other cytokines studied.

**Table 4:** The incidence of various irAEs, including the total number of patients affected by each irAE, the number of patients treated with prednisolone at the time of serum collection, and the number of patients not treated with prednisolone at the time of serum collection.

Type of irAE	Total patients	With prednisolone	Without prednisolone
Pneumonitis	15	7	8
Dermatitis	30	13	17
Hepatitis	7	1	6
Thyroiditis	8	3	5
Pancreatitis	5	2	3
Fatigue	16	3	13
Arthralgia	7	4	3
Colitis	15	9	6
Hypophysitis	3	1	2
Nephritis	4	1	3

## Discussion

In this study, we have shown that certain cytokines are associated with specific irAEs in two ways: Higherbaseline serum levels of certain cytokines are associated with future development of specific irAEs and for other cytokines an increase in serum level during the course of treatment is associated with a simultaneous occurrence of specific irAEs.

MIG is the cytokine that, at baseline, shows the strongest predictive value for several irAEs in our study. MIG and IP-10 are small cytokines belonging to the CXC chemokine family and are also known as CXCL9 (MIG) and CXCL10 (IP-10). These CXC chemokines specifically interact with the receptor CXCR3 which is nearly exclusively expressed on activated T cells (10). MIG plays a role in the induction of chemotaxis, promotion of differentiation and multiplication of leukocytes, and cause tissue extravasation, while IP-10 has pro-inflammatory and anti-angiogenic properties (11). An early increase in MIG and IP-10, 1 to 2 weeks after the start of ICI therapy, has previously demonstrated to increase the risk of developing irAEs (9). We found that high levels of IP-10 at baseline is associated with high risk of developing an allergic reaction, hepatitis, pancreatitis and dermatitis, while high levels of MIG at baseline is associated with high risk of developing arthralgia, colitis, hypophysitis, pancreatitis, thyroiditis and dermatitis. These findings appear to contradict those of a previous study where significantly lower levels of MIG and IP-10 at baseline were observed in the irAE group of patients receiving ICI, but an increase shortly after treatment (12). However, none of the patients in that study had the diagnosis MP and there is evidence to suggest that pretherapy cytokine expression can vary significantly among patients with different types of cancer (13). On the other hand, MIG has been implicated in a variety of autoimmune conditions including thyroiditis, type 1 diabetes mellitus, Addison’s disease, and inflammatory bowel disease in previous studies (14). MIG and its receptor CXCR3 have been reported as being important in the development of autoimmune thyroiditis (10) and experimental evidence also supports the concept that MIG and IP-10 and their receptor, CXCR3, play an important role in the initial stage of autoimmune disorders involving endocrine glands (14) supporting our finding of association between MIG and thyroiditis and hypophysitis. It has been shown that circulating MIG is increased in patients with atopic and contact dermatitis and increased expression of MIG and IP-10 in tissue in irAE dermatitis is also previously confirmed (15–17). This reinforces our findings that elevated levels of IP-10 and MIG are associated with dermatitis.

We found a positive association between macrophage migration inhibitory factor (MIF) and fatigue, both before and after treatment. Previous studies have shown both positive and negative association between MIF and fatigue. MIF is produced by T cells and by the pituitary gland and it can be classified as a proinflammatory cytokine as well as a hormone (18). It is involved in various functions, including leukocyte recruitment, inflammation, immune responses, cell proliferation, tumorigenesis, and counter-regulation of glucocorticoids (19). MIF is also expressed by endocrine organs involved in the stress response, especially by the pituitary gland. Pituitary-derived MIF has important roles in the periphery such as antagonizing the effects of glucocorticoids. It is well recognized that low levels of glucocorticoids, commonly referred to as stress hormones, can induce fatigue (20). This is in line with the positive association with MIF and fatigue found in our data.

**Figures 2**, **3** shows that CTACK (CCL27) is the only cytokine negatively associated with irAEs, suggesting a protective role. High baseline and treatment levels of CTACK are negatively associated with future and concurrent hepatitis, respectively. Primarily linked to immune responses in skin and mucosal tissues, CTACK recruits and activates certain T-cells (21). However, existing research does not support a central role for CTACK in hepatitis inflammation.

An elevated level of eotaxin, both before and after start of treatment, is associated with colitis and pneumonitis. These findings are consistent with previous research. Eotaxin, also known as CCL11 (C-C motif chemokine ligand 11) has chemotactic activity for eosinophils. Eotaxin is suggested to contribute to the pathogenesis of eosinophilic pneumonia through the specific recruitment of eosinophils in the lung (22). Elevated eosinophil percentage in peripheral blood has previously been shown to be associated with the diagnosis of checkpoint inhibitor-induced pneumonitis (23). With time, eotaxin has been recognized as a major mediator of intestinal inflammation and increased levels of eotaxin have been described in inflammatory bowel disease (IBD) including ulcerative colitis and Crohn’s disease (24, 25).

While prednisolone is known for its anti-inflammatory properties, its minimal impact on cytokine levels in our research is understandable. Given that most patients had only recently begun prednisolone treatment before serum samples were collected and were still exhibiting symptoms at the time of collection, the minimal effect of prednisolone can be explained by the fact that cytokine levels indicate ongoing biological activity despite the anti-inflammatory treatment. Furthermore, the number of patients analyzed was limited (**Table 4**).

To our knowledge, this is the first study to report associations between serum cytokine changes and irAEs in patients with PM treated with ICI. While many findings align with earlier research done in patients with other cancer types, some appear inconsistent. These findings highlight the complexity of the relationship between baseline cytokine levels and the development of irAEs. Serum levels of cytokines are affected by multiple factors and the association with irAEs may be influenced by the type of ICI used, the underlying diagnosis and the patient’s individual immune system.

Our analysis was limited by missing time points for some patients, preventing the use of all time information after treatment and limiting the robustness of longitudinal analyses. While the use of generalized linear mixed models helped account for this variability in observation numbers, we recognize that this may impact the interpretation of cytokine trends over extended treatment periods.A potential selection bias may exist due to the focus on patients with irAEs in the last four assay batches. We addressed this in our analysis by employing generalized linear mixed models, which include data from both patients with and without irAEs and account for cytokine levels across time points. While this approach mitigates some variability, we acknowledge that this selection could still influence our results, and we recognize it as a limitation of the study. Additionally, we did not jointly analyze multiple irAEs for the same patients due to limitations of the used statistical models Another limitation of our study is the small number of patients experiencing certain specific irAEs, such as hypophysitis (n = 4), pancreatitis (n = 5), and nephritis (n = 4). These small numbers reduce the statistical power of our analyses, limiting our ability to detect robust associations between cytokine levels and these rare irAEs. This means that some cytokine associations may be missed or underestimated, particularly for rarer irAEs. While we observed trends in cytokine dynamics for these events, larger studies will be needed to confirm and validate these findings. Future research with more substantial patient numbers will help to increase the reliability and generalizability of the identified biomarkers.

## Conclusion

We found that elevated baseline levels of MIG predicts future development of multiple irAES, while the association with ongoing irAEs (diagnostic value) is lacking or weak. Other cytokines, such as eotaxin, MIP-1α, IP-10, TNF-α, CTACK, MIP-1β, IL-4, MIF, IL-16, IL-2RA, SCGF.β, and PDFG-BB are also associated with increased risk of developing certain irAEs. Increased levels of eotaxin, CTACK, MIF, TNF-α, MIP-1β, IL-4, and IL-16, during treatment are associated with specific irAEs. MIF is identified as both a predictive and diagnostic marker for fatigue. Secretion of these cytokines may serve as predictive and diagnostic biomarkers for irAEs. Validation in larger cohorts would be of interest, particularly for associations like MIF with fatigue, eotaxin with colitis and pneumonitis, MIG with endocrine inflammation, and MIG and IP-10 with dermatitis. These findings align with existing research and suggest their potential as diagnostic biomarkers.

## Data Availability

The raw data supporting the conclusions of this article will be made available by the authors, without undue reservation.
