# Supplementary figures and images for: Serum cytokines as a biomarker for immune checkpoint inhibitor toxicity in patients with pleural mesothelioma
Source: Front Immunol. 2024 Dec 2;15:1480183. doi: 10.3389/fimmu.2024.1480183 (PMC11647018; doi:10.3389/fimmu.2024.1480183)

# Thyroiditis

log2 fold change

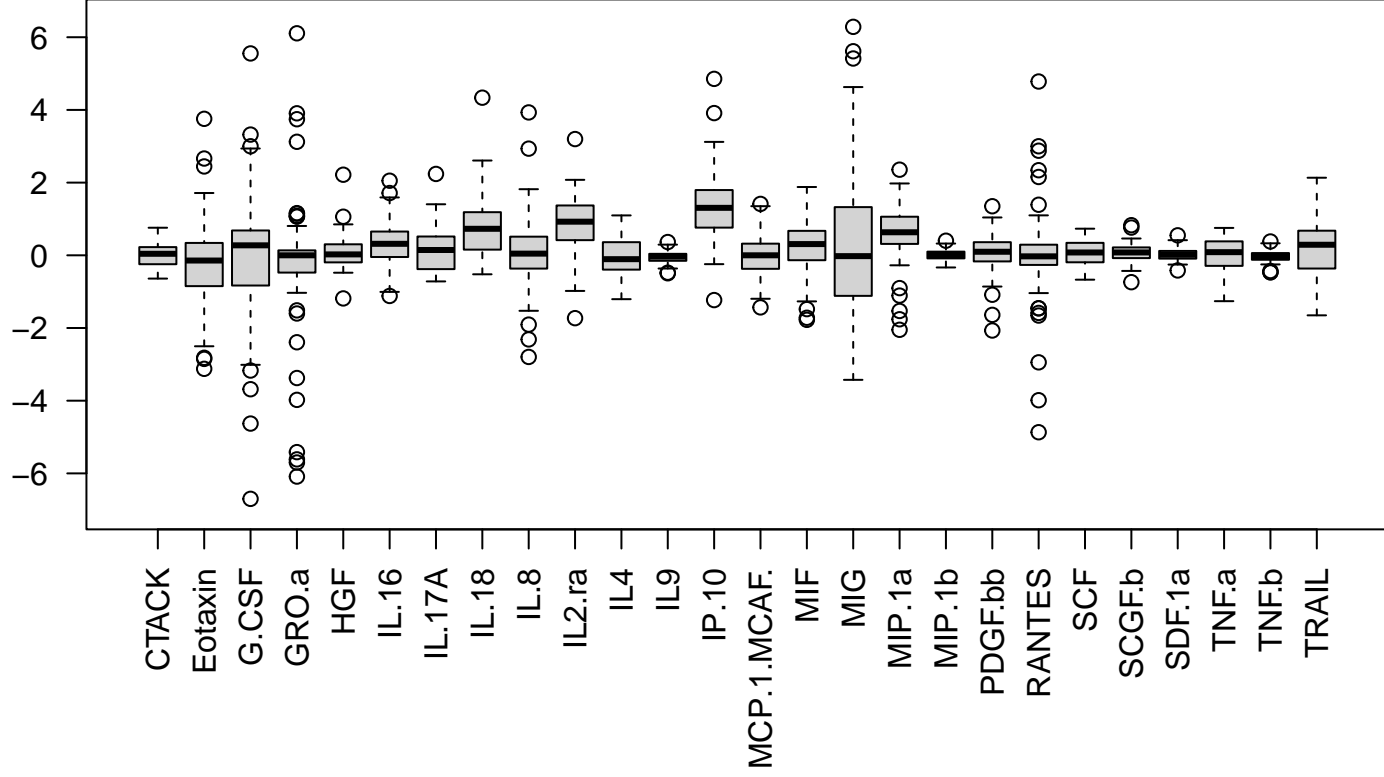

Supplement: Supplementary file 1 [file DataSheet1.pdf]
