# Supplementary material for: Serum cytokines as a biomarker for immune checkpoint inhibitor toxicity in patients with pleural mesothelioma
Source: Front Immunol. 2024 Dec 2;15:1480183. doi: 10.3389/fimmu.2024.1480183 (PMC11647018; doi:10.3389/fimmu.2024.1480183)

**Supplements**

Mixed models for analyzing cytokines.

We can visualize the selection frequency of all cytokines. The left panel barplot shows the identified cytokines, identified at least 10 out of 100 times. The right panel shows the mean effect with error bar as standard deviation. The upper section illustrates the Cytokine:Time interaction, association between cytokine level and an ongoing irAE at any given point in time. The lower section is the associations between baseline cytokine levels and future development of irAEs.

**Allergic reaction**


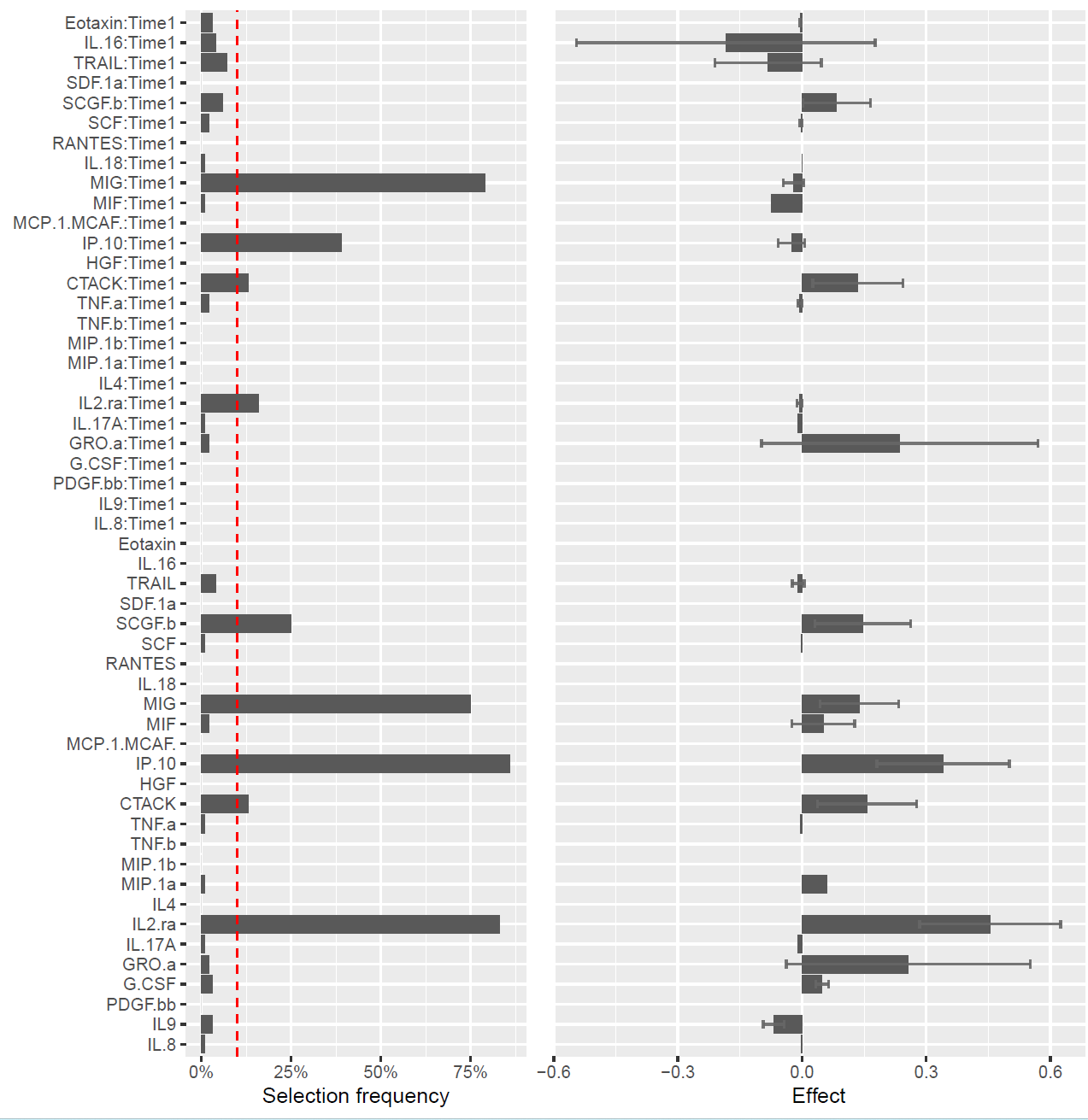


**Thyroiditis**
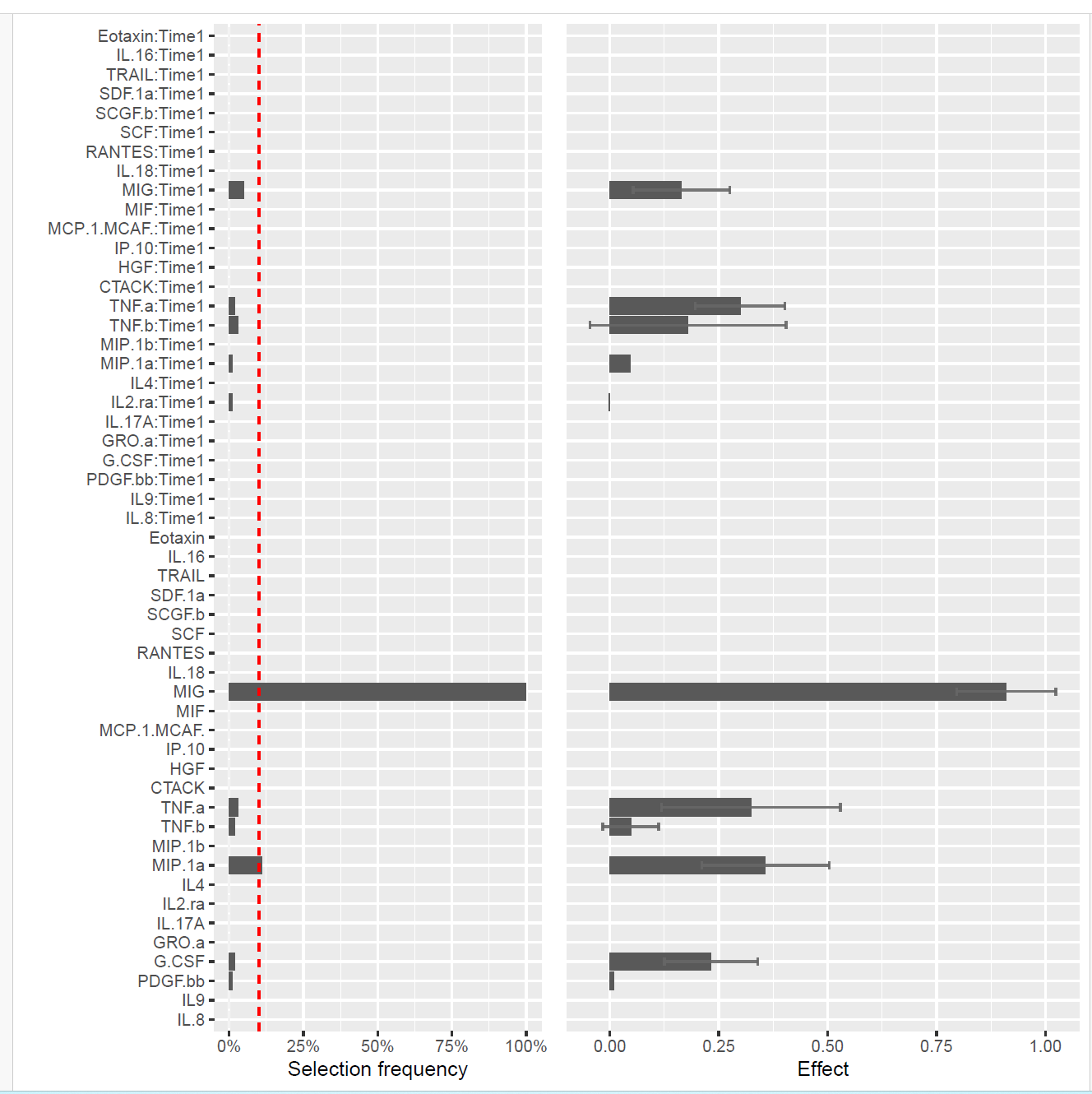


**Pneumonitis**


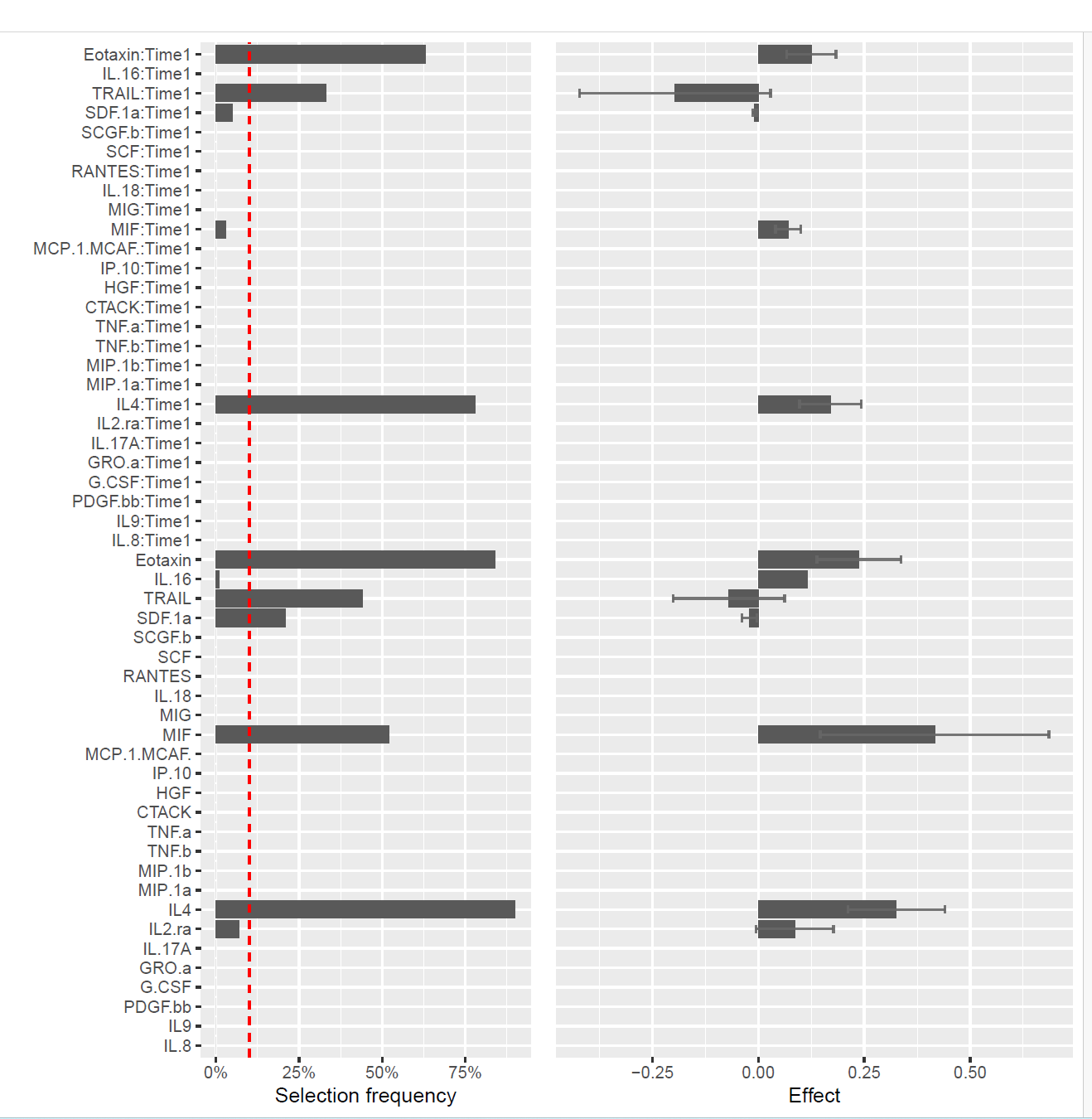


**Nephritis**


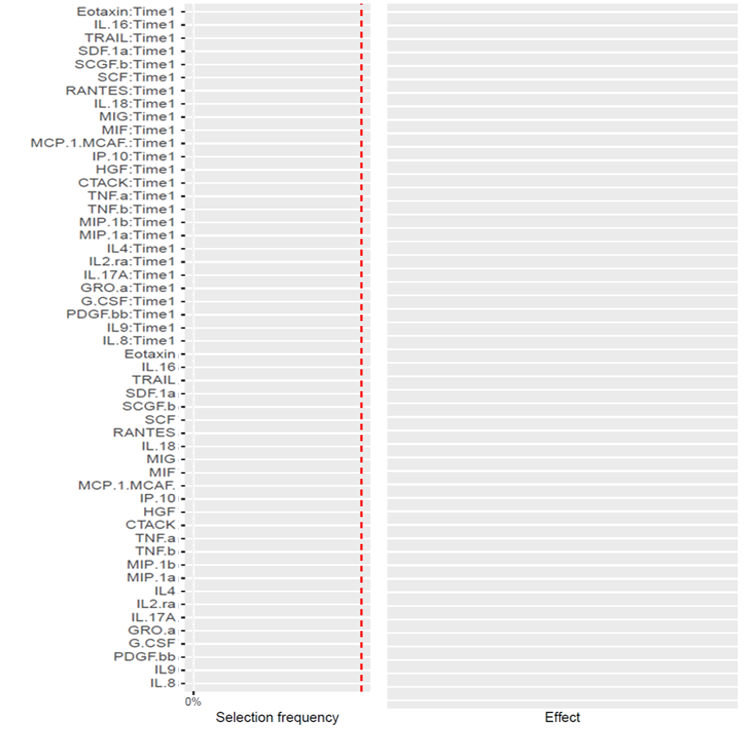


**Pancreatitis**


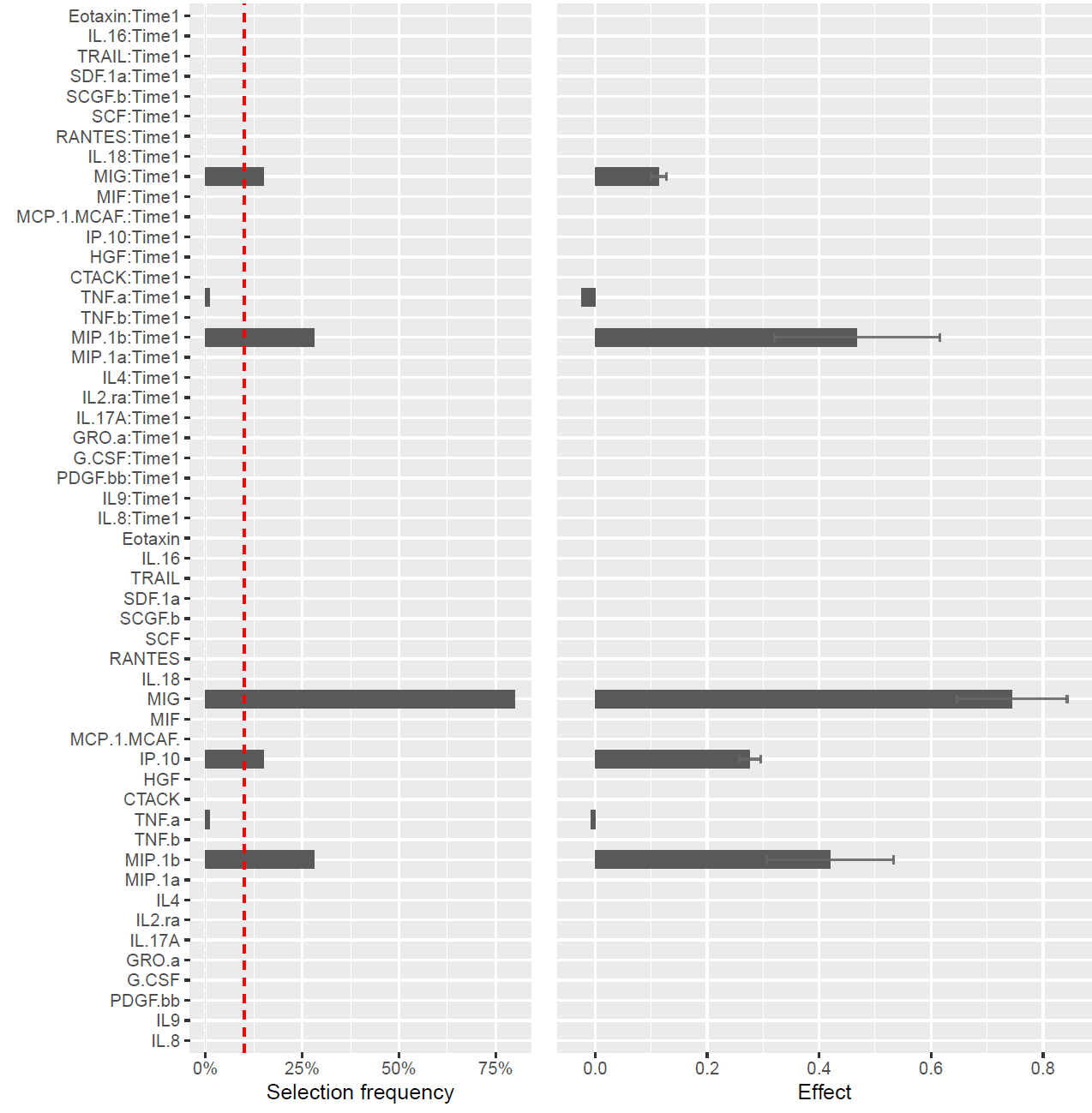


**Hypophysitis**


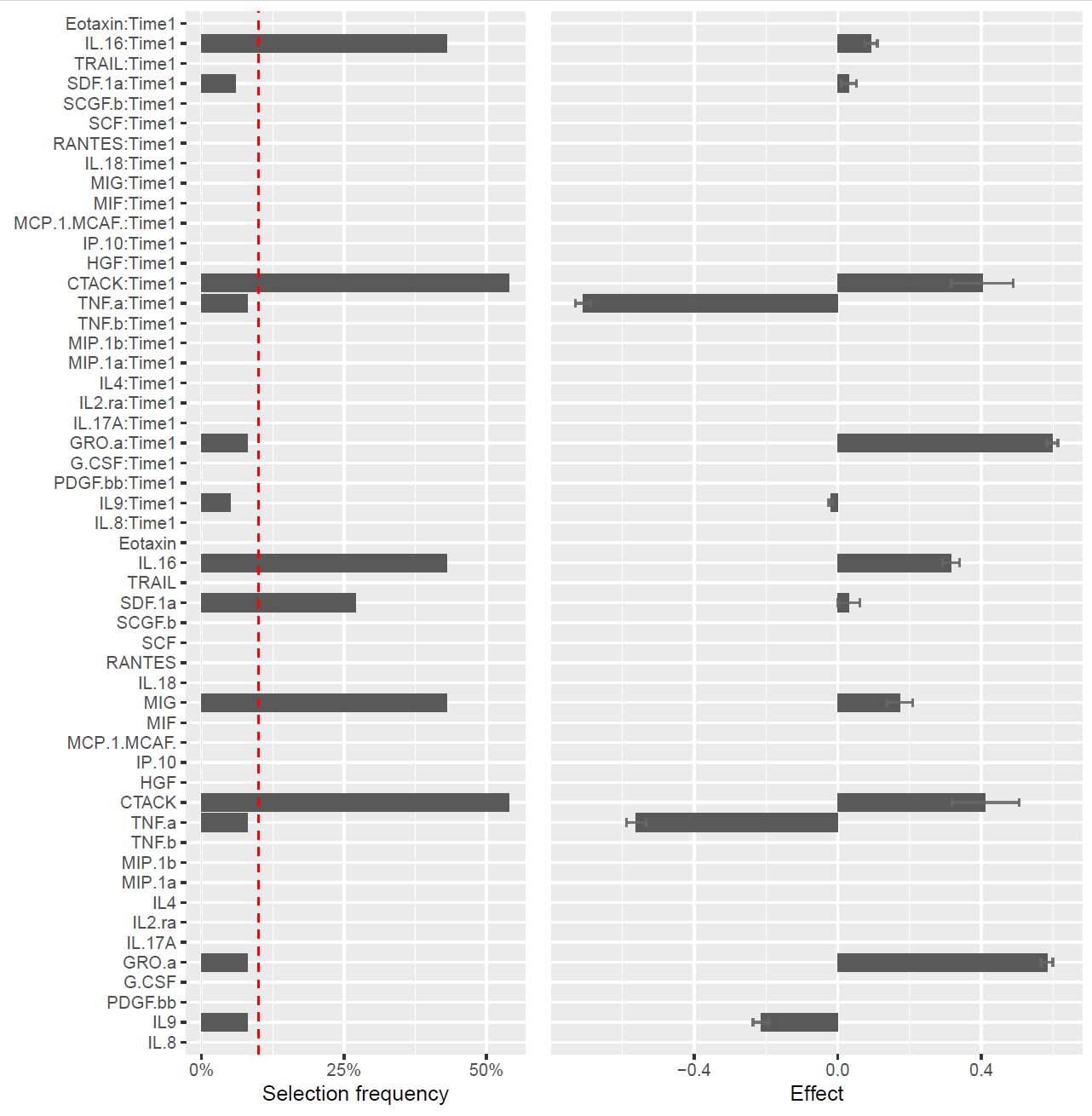


**Hepatitis**


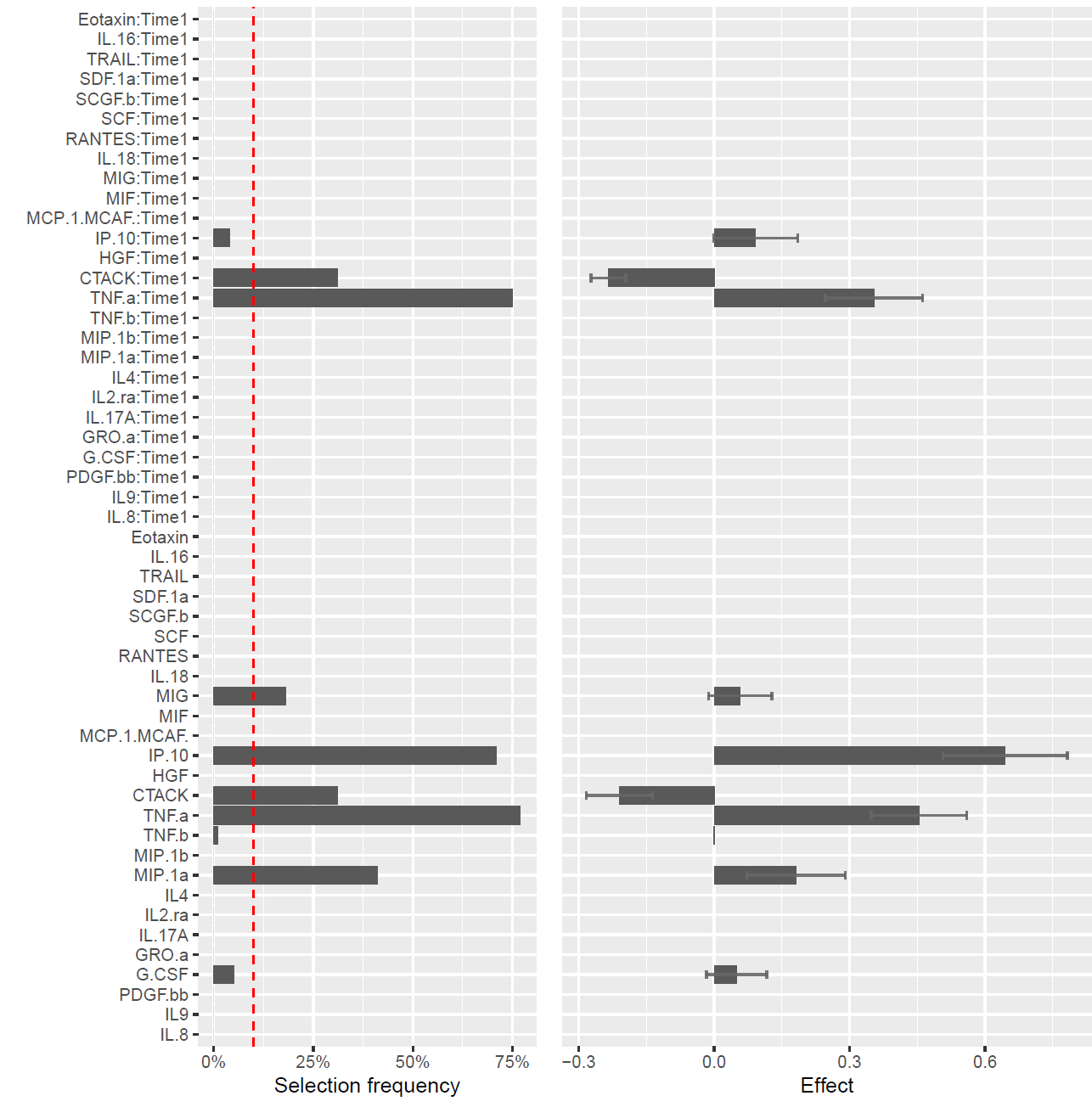


**Fatigue**


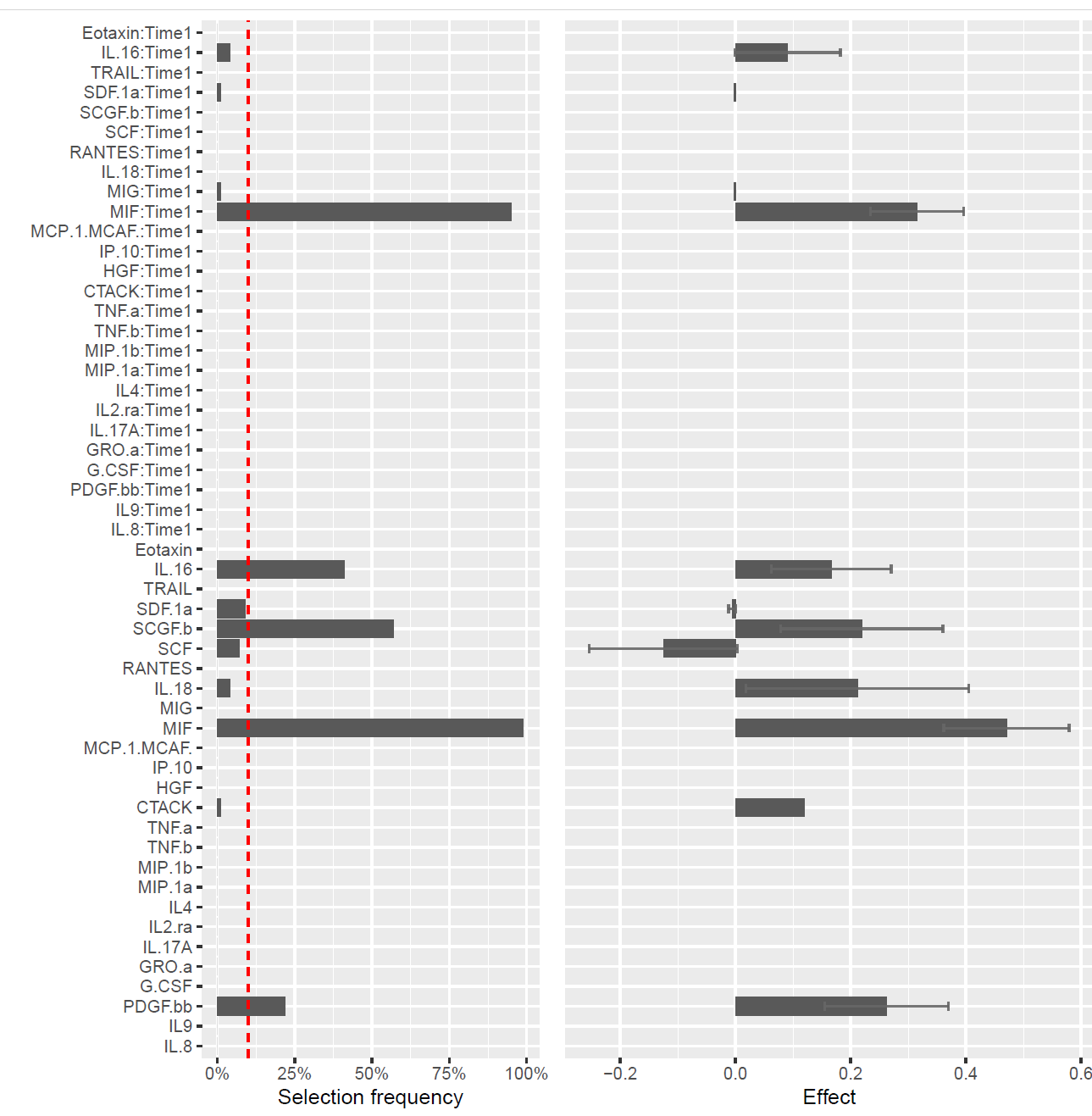


**Dermatitis**


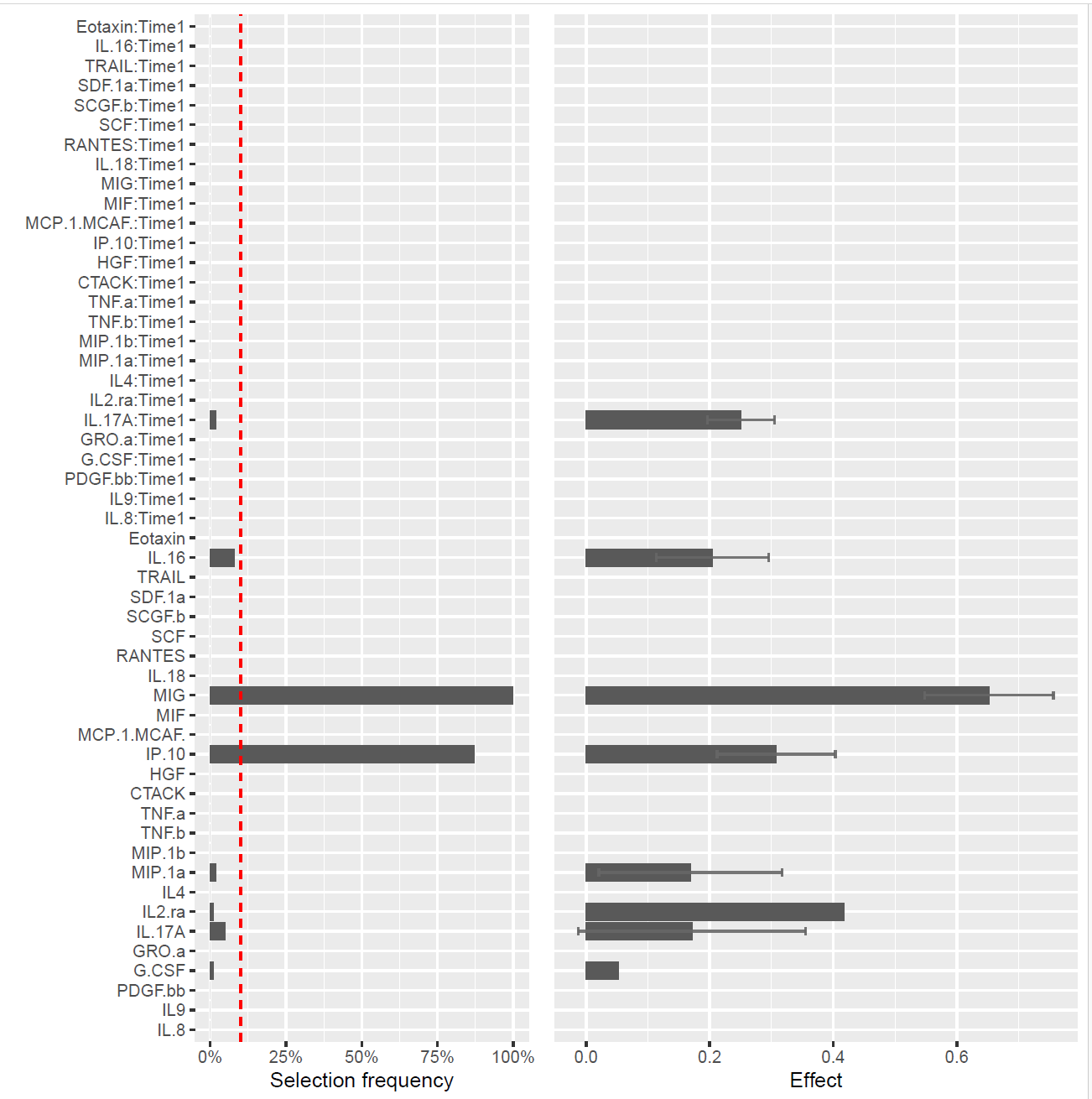


**Colitis**


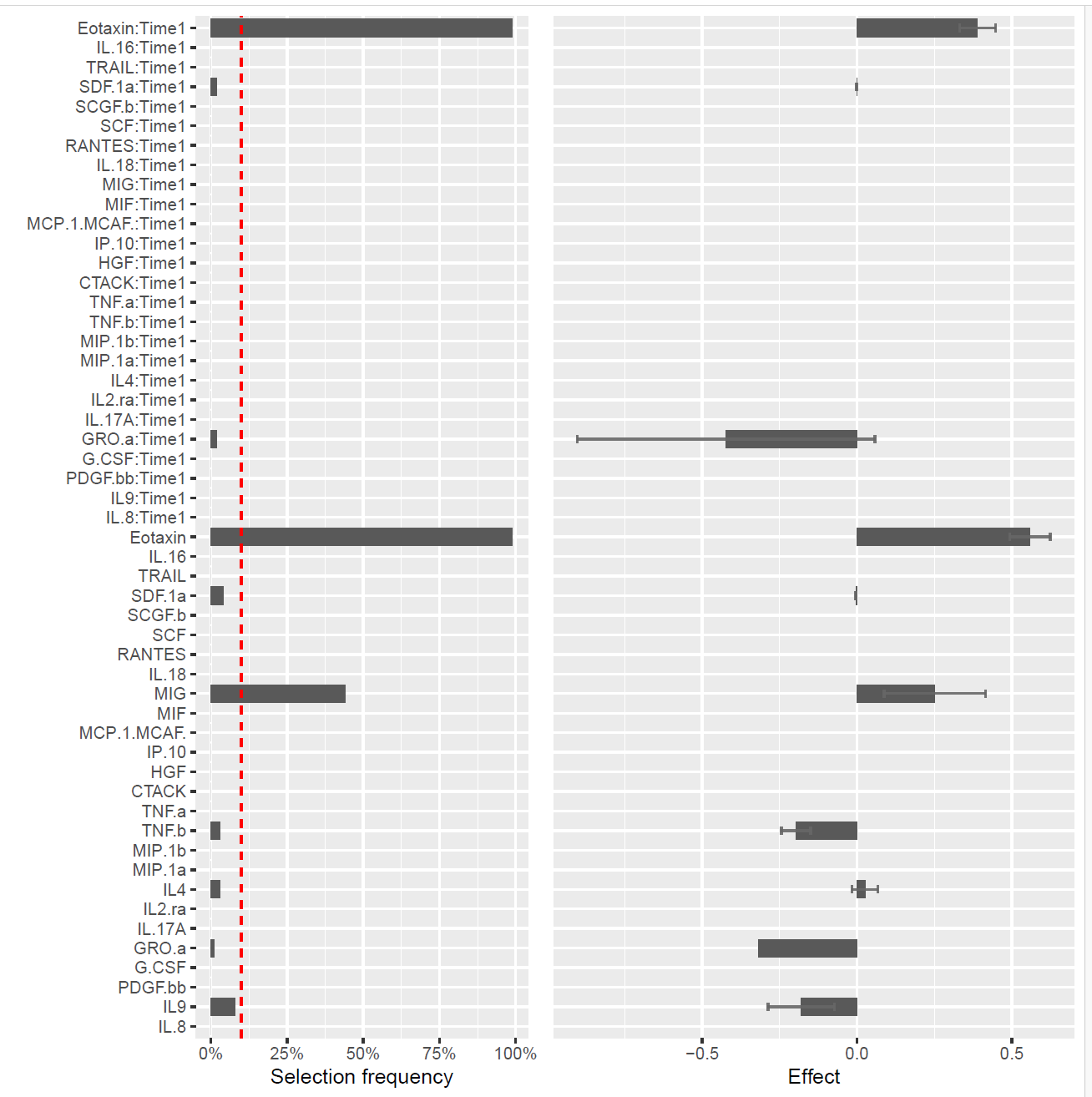


**Arthralgia**


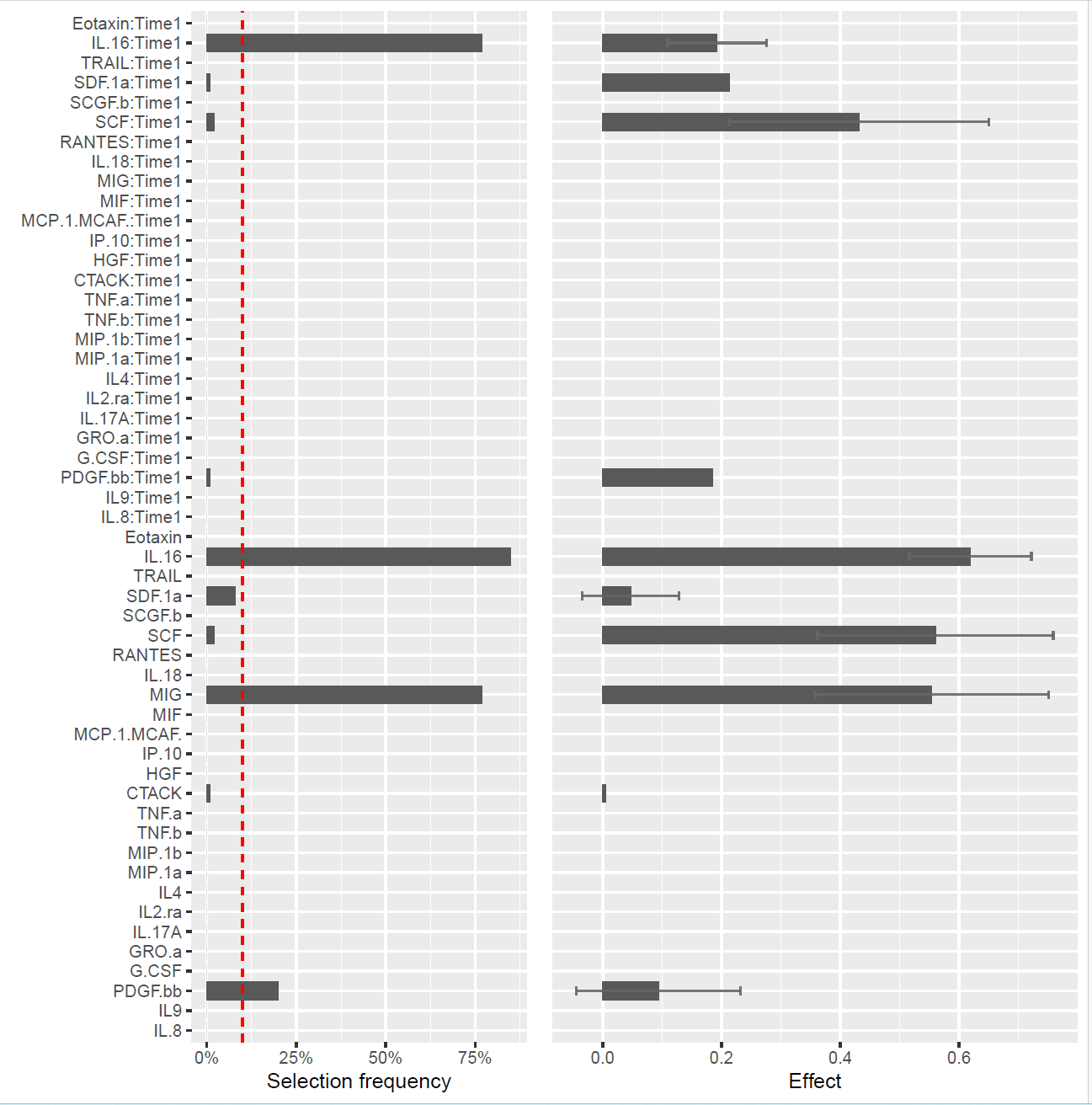

Supplement: Supplementary file 2 [file Table1.docx]
